# Outcome of Percutaneous Coronary Intervention for Left Versus Non-left Main Coronary Artery in Acute Coronary Syndrome: A Comparative Study

**DOI:** 10.7759/cureus.91368

**Published:** 2025-08-31

**Authors:** S M Mamun Iqbal, A M Shafique, Neuton Mondal, Mohammad Jahid Hasan

**Affiliations:** 1 Department of Cardiology, MH Samorita Hospital and Medical College, Dhaka, BGD; 2 Department of Cardiology, United Hospital Limited, Dhaka, BGD; 3 Department of Health System Research, Tropical Disease and Health Research Center, Dhaka, BGD

**Keywords:** acute coronary syndromes (acs), cardiac death, left main coronary artery (lmca), major adverse cardiovascular events (mace), percutaneous coronary intervention (pci)

## Abstract

Background and objective: Acute coronary syndromes (ACS), particularly those affecting the left main coronary artery (LMCA), are associated with high morbidity and mortality. The objective of the present study was to investigate long-term outcomes in patients who underwent percutaneous coronary intervention (PCI) for ACS affecting the LMCA compared to those with non-LMCA involvement.

Methods: This interventional study was conducted at the Department of Cardiology of MH Shamorita Medical College Hospital in Dhaka, Bangladesh, from January 2023 to June 2024. A total of 101 patients with ACS who underwent PCI participated in the study. Of whom, 51 were in the LMCA group and 50 were in the non-LMCA group. Follow-up assessment was done at the third, sixth, and 12th months, focusing on major adverse cardiovascular events (MACE) as the primary endpoint and persistent symptoms, repeat revascularization, and stent thrombosis as secondary endpoints.

Results: The mean age of the LMCA and non-LMCA groups was 56 and 54 years, respectively, with a higher female proportion in the LMCA group. The patients in the LMCA group presented with more complex lesions, with a mean synergy between percutaneous coronary intervention with Taxus and cardiac surgery (SYNTAX) score of 23.6. The MACE rate was notably higher in the LMCA group at 6%, compared to 2% in the non-LMCA group (p=0.013). Myocardial infarction (MI) occurred in 4% of the LMCA group, with no events in the non-LMCA group. Persistent symptoms and repeat revascularization were also more prevalent in the LMCA group (6% and 4%, respectively, p-value <0.05).

Conclusions: Patients with ACS involving the LMCA experienced higher rates of adverse outcomes, particularly MI and repeat revascularization, following PCI compared to non-LMCA cases.

## Introduction

Acute coronary syndrome (ACS) represents a spectrum of cardiovascular conditions, such as ST-segment elevation myocardial infarction (STEMI), non-ST-elevation myocardial infarction (NSTEMI), and unstable angina, characterized by a sudden compromise in blood flow to the heart due to blockage or narrowing of coronary arteries secondary to ruptured atherosclerotic plaque and thrombus formation [1]. Among the coronary arteries, the left main coronary artery (LMCA) supplies a significant area of the left ventricle through the left anterior descending (LAD) and circumflex arteries [2]. Hence, occlusion of the LMCA often leads to more extensive myocardial infarctions (MIs), causing severe ischemia and left ventricular dysfunction. As a result, ACS involving the LMCA typically carries a worse prognosis than ACS involving non-LMCA coronary arteries and accounts for a significant proportion of fatal cardiac events [3]. Epidemiological data suggest that LMCA involvement occurs in approximately 3% to 10% of patients presenting with ACS but is associated with disproportionately high morbidity and mortality due to the large myocardial territory at risk [4].

The high mortality in LMCA-related ACS is primarily attributed to the rapid deterioration of cardiac function, leading to multisystem organ failure if left untreated. Immediate and effective intervention is paramount in managing these cases. Historically, surgical revascularization through coronary artery bypass grafting (CABG) was the mainstay treatment for LMCA disease [5]. However, with the advancement of percutaneous coronary intervention (PCI), this less invasive approach has become a viable and increasingly preferred option for many patients, especially in the acute setting of ACS [6]. However, in acute ACS settings, especially in resource-limited environments like Bangladesh, CABG may not be feasible due to delays, limited surgical capacity, and patient comorbidities. Therefore, PCI is often preferred as a timely and life-saving intervention, enabling rapid myocardial reperfusion.

Current clinical guidelines for the management of ACS, including both STEMI and NSTEMI, recommend emergency revascularization through primary PCI, particularly for patients presenting with cardiogenic shock [7]. In fact, PCI has become the main modality of reperfusion therapy in ACS, offering rapid restoration of coronary blood flow and reducing myocardial damage. The benefit of PCI in patients with cardiogenic shock is clear; emergency coronary reperfusion through this method is supported by robust evidence as a lifesaving intervention [8]. Prior studies have reported comparable challenges with LMCA PCI, including higher rates of MACE and procedural complexity, but data on long-term outcomes remain scarce [9-12].

Patients undergoing PCI for LMCA disease often face more complex interventions due to the anatomical and clinical challenges associated with the artery. The bifurcation of the LMCA into the LAD and circumflex arteries introduces technical difficulties, which can complicate stenting and increase the likelihood of restenosis or stent thrombosis [6]. Furthermore, LMCA lesions are often more calcified and diffuse, requiring advanced PCI techniques, such as rotational atherectomy or intravascular imaging guidance [6,7]. These complexities make PCI for LMCA disease a high-risk procedure, and concerns remain about long-term complications such as repeat revascularization, restenosis, and mortality [13]. Comparing LMCA to non-LMCA PCI outcomes in ACS specifically (rather than stable CAD) is clinically relevant because acute plaque rupture and thrombosis drive distinct procedural risks, ischemic burden, and post-PCI complications that differ from elective interventions in stable disease.

However, there is a scarcity of evidence regarding the long-term outcomes of PCI in patients with ACS involving LMCA in comparison to non-LMCA, particularly in the context of a resource-constrained setting like in Bangladesh. This study addresses knowledge gaps by evaluating real-world procedural outcomes, completeness of revascularization, and MACE in LMCA versus non-LMCA ACS, thereby providing novel data relevant to low-resource settings where CABG may not be immediately available. The objective of the present study is to compare the long-term outcomes of PCI in patients with ACS involving the LMCA versus non-LMCA, focusing on major adverse cardiovascular events (MACE: MI, stroke, or sudden cardiac death) and additional outcomes including persistent angina, dyspnea, stent thrombosis, target vessel revascularization, and complete treatment of all significant lesions.

*This article was previously presented as a meeting abstract at the 2025 Transcatheter Cardiovascular Therapeutics Asia Pacific (TCTAP) annual cardiovascular summit in Korea on April 25, 2025*.

## Materials and methods

Study design and setting

This is an interventional study conducted in the Department of Cardiology of MH Samorita Medical College and Hospital, a tertiary care hospital situated in Dhaka, Bangladesh, from January 2023 to June 2024. The Institutional Ethical Review Board of MH Samorita Medical College approved the study (approval no. MHSHMC/IERB_

Inclusion and exclusion criteria

All patients admitted to the study hospital during the specified time period with a diagnosis of ACS, including STEMI, NSTEMI, and unstable angina, constituted the population for the present study. From this population, participants were selected based on predetermined inclusion and exclusion criteria and divided into two distinct groups. Patients were included if they were aged ≥18 years, diagnosed with ACS, and underwent PCI for a culprit lesion. Patients were excluded if they had prior CABG, cardiogenic shock requiring inotropes or mechanical support at presentation, severe comorbidities limiting PCI (e.g., advanced renal failure, severe hepatic dysfunction, or active infection), contraindications to antiplatelet therapy, or declined consent for the procedure. The first group comprised patients diagnosed with ACS with a suspected culprit lesion involving the LMCA who underwent PCI. The second group included those diagnosed with ACS and suspected culprit lesions in coronary arteries other than the LMCA (non-LMCA) who also underwent PCI. Patients who did not undergo PCI were excluded from the study, either because they did not meet PCI eligibility criteria or declined consent for the procedure. In total, 101 patients were included in the study, with 51 in the LMCA group and 50 in the non-LMCA group.

A consecutive sampling method was used to select participants. This approach was chosen due to logistical considerations, including the limited study duration and the need to include all eligible cases as they presented, ensuring comprehensive and unbiased data collection. As consecutive sampling was done, no a priori sample size calculation was performed. However, the relatively low frequency of observed adverse events limited the ability to detect statistically significant differences between groups for rare outcomes. To address this, a post-hoc power analysis was conducted and is presented in the results section.

Study procedure

Upon admission, all patients diagnosed with ACS were screened for eligibility to undergo PCI. Those meeting the inclusion criteria and providing written informed consent were enrolled in the study. All patients underwent PCI based on standard clinical indications for ACS, including STEMI, NSTEMI, or unstable angina with angiographically significant lesions. The choice of treatment strategy, such as single-stent versus two-stent approaches and provisional versus upfront bifurcation techniques, was guided by lesion anatomy, severity, bifurcation involvement, and hemodynamic stability. The diagnoses of STEMI, NSTEMI, and unstable angina were based on standardized criteria, including clinical presentation, ECG changes, and cardiac biomarker elevation, ensuring consistent classification across all participants.

Before the procedure, all patients received aspirin (300 to 325 mg) at least two hours before PCI, followed by a maintenance dose of 75 mg daily indefinitely, consistent with clinical guidelines. A loading dose of clopidogrel, prasugrel, or ticagrelor was administered before PCI, with continued therapy for at least one year. High-intensity statin preloading with atorvastatin (80 mg) or rosuvastatin (40 mg) was given to reduce periprocedural myocardial injury. Anticoagulation was primarily achieved using unfractionated heparin, with bivalirudin or low molecular weight heparin used as alternatives when clinically indicated.

All PCI procedures were performed by a single experienced operator following standard protocols [3]. Lesion complexity, including SYNTAX score, was assessed pre-procedurally for both LMCA and non-LMCA groups. Lesion classification (e.g., bifurcation, ostial, distal) and calcification severity were determined by angiographic assessment by the operator and confirmed by a second independent cardiologist through review of imaging records. Vascular access was obtained via the radial or femoral artery, chosen based on operator preference, patient anatomy, and clinical status. Angiographic assessment defined lesion characteristics, including severity, location, and bifurcation involvement. In patients with LMCA disease who also had lesions in the left anterior descending (LAD) or left circumflex (LCX) arteries, treatment was planned from distal to proximal lesions, unless the LMCA stenosis was critical (>90%) or the patient was unstable, in which case LMCA treatment was prioritized. All LMCA cases included in the study were unprotected, reflecting the acute presentation of these patients; protected LMCA cases (with patent bypass grafts) were excluded to maintain homogeneity in procedural risk assessment.

Lesion preparation was done by pre-dilatation using compliant balloons, with scoring or cutting balloons employed in heavily calcified or resistant lesions to optimize vessel compliance and facilitate stent delivery. Drug-eluting stents were predominantly used, and critical LMCA lesions were treated with balloon angioplasty followed by stent implantation. For distal LMCA bifurcation lesions, a provisional single-stent technique was preferred, with a second stent deployed if needed due to side branch compromise or suboptimal initial results. Upfront two-stent strategies such as mini-crush, T and small protrusion (TAP), or V-stenting were employed based on anatomical complexity. Following stent deployment, high-pressure post-dilatation was performed to ensure optimal stent apposition, and kissing balloon inflation was routinely applied after two-stent procedures to optimize bifurcation stent expansion and vessel patency. Intracoronary imaging modalities such as intravascular ultrasound or optical coherence tomography were not routinely used but were available at the operator's discretion. Decisions regarding LMCA PCI were made by the operating interventional cardiologist. A formal Heart Team consultation was not routinely sought due to the limited availability of a multidisciplinary team.

Throughout the procedure, patients were closely monitored for hemodynamic stability and ECG changes. Procedural success was defined as residual stenosis less than 20% with thrombolysis in myocardial infarction (TIMI) flow grade 3 and absence of major complications. Improvement in TIMI flow was assessed by the operating interventional cardiologist during the procedure and documented in the procedural report. Any procedural complications, such as coronary dissection, no-reflow phenomenon, or access site complications, were recorded and managed per institutional protocols. Post-procedure, patients were monitored in a coronary care unit for at least 24 hours. Aspirin was continued indefinitely at 75 mg daily, and dual antiplatelet therapy (such as including aspirin and a P2Y12 inhibitor like clopidogrel) was maintained for at least one year to reduce stent thrombosis risk. Besides, high-intensity statins, beta-blockers, and angiotensin-converting enzyme (ACE) inhibitors or angiotensin receptor blockers (ARBs), tailored to individual clinical indications. Secondary prevention therapies, including statins, were prescribed according to current guidelines [14].

Follow-up

All patients were closely monitored following their PCI, with follow-up evaluations scheduled at three months, six months, and 12 months after the procedure. These follow-up assessments were conducted either through in-person clinic visits or via telephone interviews, depending on the patient’s circumstances and clinical needs. Data regarding symptoms like persistent angina and shortness of breath, or dyspnea, as well as other outcome variables, were collected. Patients received guideline-directed medical therapy throughout the follow-up period, including dual antiplatelet therapy (aspirin plus a P2Y12 inhibitor, such as clopidogrel), statins, beta-blockers, and ACE inhibitors or ARBs, tailored to individual clinical indications. Any modifications to therapy, adherence issues, or adverse drug effects were documented at each visit. No patients were lost to follow-up, and all outcome data were complete for analysis, eliminating the need for imputation or exclusion due to missing data.

Endpoints

The primary endpoint of the present study was the occurrence of MACE. Major adverse cardiovascular events are defined as a composite of MI, stroke, and sudden cardiac death [15]. Myocardial infarction was defined according to the Fourth Universal Definition of MI; stroke was defined as a sudden neurological deficit lasting >24 hours confirmed by imaging; and sudden cardiac death was defined as unexpected death occurring within one hour of symptom onset or unwitnessed death in a previously stable patient. All events were adjudicated by two independent cardiologists based on clinical records, laboratory results, and imaging studies. Additionally, persistent angina, persistent shortness of breath or dyspnea, the requirement of target vessel revascularization, and stent thrombosis were considered as secondary endpoints of the present study. Complete revascularization was defined as successful PCI of all angiographically significant lesions (≥70% stenosis in major epicardial vessels or ≥50% in the left main) identified at the time of the index procedure or planned staged procedures. The assessment of complete revascularization was based on post-PCI angiography and documented in the procedural reports. Secondary endpoints were assessed during hospital stay and follow-up visits and adjudicated by the same cardiology team using patient interviews, medical records, and angiographic findings. This study was not registered in a clinical trial registry because it was conducted as an observational study based on routine clinical practice without any experimental intervention.

Statistical analysis

The statistical analysis was performed using SPSS Statistics (IBM Corp., Armonk, NY, USA). Appropriate statistical tests were carried out to assess the differences in baseline characteristics and outcomes between the LMCA and non-LMCA groups. Continuous variables were expressed as mean with standard deviation (SD) and compared using either the Student’s t-test or the Wilcoxon rank test, depending on the distribution of the data. Categorical variables were expressed as percentages and compared using the chi-squared test or Fisher’s exact test. All variables included in the analysis were complete for all patients, ensuring that all outcomes could be assessed without exclusion. Due to the relatively small sample size and low frequency of adverse events, multivariable regression analyses were not performed; baseline characteristics, including hypertension, diabetes, and family history, were carefully compared, and any significant differences were considered when interpreting outcomes. For all statistical analyses, a two-sided p-value of <0.05 was considered to indicate statistical significance.

## Results

Baseline characteristics

The average age was 56 years for the LMCA group and 54 years for the non-LMCA group. Notably, the LMCA group had a higher prevalence of female patients (31% vs. 14%) and comorbidities, including hypertension (76% vs. 54%) and family history of coronary artery disease (27% vs. 4%) (Table 1).

**Table 1:** Baseline characteristics of the patients (total n = 101) LMCA: Left main coronary artery, PCI: Percutaneous coronary intervention

Characteristics	LMCA, n = 51	Non-LMCA, n = 50	Test statistic (value)	p-value
Age	56.06 (11.23)	54.14 (10.58)	t = 0.94	0.350
Age group			χ² = 0.62	0.430
<60	27 (52.94)	30 (60.00)		
>60	24 (47.06)	20 (40.00)		
Sex			χ² = 5.97	0.015
Female	16 (31.37)	7 (14.00)		
Male	35 (68.63)	43 (86.00)		
Comorbidities				
Type 2 diabetes mellitus	29 (56.86)	23 (46.00)	χ² = 1.17	0.280
Hypertension	39 (76.47)	27 (54.00)	χ² = 5.77	0.016
Dyslipidemia	8 (15.69)	5 (10.00)	χ² = 0.74	0.390
Chronic kidney disease	7 (13.73)	4 (8.00)	χ² = 0.87	0.350
Chronic respiratory diseases	3 (5.88)	4 (8.00)	χ² = 0.21	0.650
Family history of coronary artery disease	14 (27.45)	2 (4.00)	χ² = 9.21	0.002
Smoking tobacco	4 (8.00)	0 (0.00)	Fisher’s exact test	0.117
Smokless tobacco consumption	2 (4.00)	0 (0.00)	Fisher’s exact test	0.493
Previous history of PCI	5 (9.80)	1 (2.00)	Fisher’s exact test	0.206
Left ventricular failure	8 (16.67)	4 (8.00)	χ² = 1.57	0.210

Clinical presentation and angiographic findings

Among the groups, STEMI was more prevalent in the non-LMCA group (24% vs. 16%), while unstable angina was more common in the LMCA group (55% vs. 42%). The mean SYNTAX score was significantly higher in the LMCA group (23.6 vs. 7.8). A higher number of patients in the LMCA group had multiple-vessel disease (39% vs. 22%), and lesions were more likely to be classified as type B (53% vs. 16%). The proportion of patients with severe calcification was notably higher in the LMCA group (49% vs. 20%). However, left ventricular ejection fraction (LVEF) and serum creatinine levels did not significantly differ between groups (Table 2).

**Table 2:** Clinical presentations of the patients (total n = 101) STEMI: ST-segment elevation myocardial infarction, NSTEMI: Non-ST-elevation myocardial infarction, UA: Unstable angina, SYNTAX: Synergy between percutaneous coronary intervention with Taxus and cardiac surgery, LMCA: Left main coronary artery, AHA: American Heart Association, ACC: American College of Cardiology, TIMI: Thrombolysis in myocardial infarction, LVEF: Left ventricular ejection fraction

Characteristics	LMCA, n = 51	Non-LMCA, n = 50	Test statistic (value)	p-value
Diagnosis			χ² = 6.92	0.031
STEMI	8 (15.68)	12 (24.00)		
NSTEMI	15 (29.41)	17 (34.00)		
UA	28 (54.90)	21 (42.00)		
SYNTAX score	23.56 (7.08)	7.78 (5.29)	t = 15.98	<0.001
Unprotected LMCA	51 (100.00)	-	-	-
Number of vessels involved			χ² = 5.85	0.016
Single	30 (60.78)	39 (78.00)		
Multiple	20 (39.22)	11 (22.00)		
Type of lesion (AHA/ACC classification)			χ² = 23.84	<0.001
A	4 (7.84)	26 (52.00)		
B	27 (52.94)	8 (16.00)		
C	20 (39.22)	16 (32.00)		
Pre-procedure TIMI flow			χ² = 3.29	0.349
0	16 (31.37)	13 (26.00)		
1	31 (60.78)	27 (54.00)		
2	3 (5.88)	8 (16.00)		
3	1 (1.96)	2 (4.00)		
LVEF %	53.90 (11.02)	55.26 (9.71)	t = 1.44	0.153
Severe calcification	25 (49.02)	10 (20.00)	χ² = 8.74	0.003
Serum creatinine level	1.10 (0.72)	1.00 (0.22)	t = 0.32	0.751

Procedural characteristics

The choice of access route significantly differed between the groups, with femoral access used in 47% of LMCA patients versus only 8% in non-LMCA patients. The majority of patients with multivessel disease in both groups underwent procedures without staged interventions. In terms of procedural strategy, most patients received a single stent strategy (82% for LMCA vs. 94% for non-LMCA). When double stents were considered for bifurcation lesions, several techniques of stenting were utilized, including the mini crush (7.84% for LMCA), TAP (5.88% for LMCA vs. 3.00% for non-LMCA), and V-stenting (1.96% for LMCA). But no significant differences were observed in their application (Table 3). Procedural complications, including coronary dissection, no-reflow, or perforation, were monitored in all patients, and none occurred in either the LMCA or non-LMCA groups.​​​​​​

**Table 3:** Procedural characteristics of the patients (n = 101)

Characteristics	LMCA, n = 51	Non-LMCA, n = 50	Test statistic (value)	p-value
Route of procedure			χ² = 22.3	<0.001
Femoral	24 (47.06)	4 (8.00)		
Radial	27 (52.94)	46 (92.00)		
Staged procedure			χ² = 0.80	0.371
No	48 (94.12)	44 (88.00)		
yes	3 (5.88)	6 (12.00)		
Type of procedure			χ² = 4.52	0.033
Double stent Strategy	9 (17.65)	3 (6.00)		
Single stent strategy	42 (82.35)	47 (94.00)		
Double stent Technique			Fisher’s exact test	0.619
Mini Crush	4 (7.84)	0 (0.00)		
TAP	3 (5.88)	3 (6.00)		
V-Stenting	1 (1.96)	0 (0.00)		

Outcomes and post-hoc power analysis

**Figure 1:**
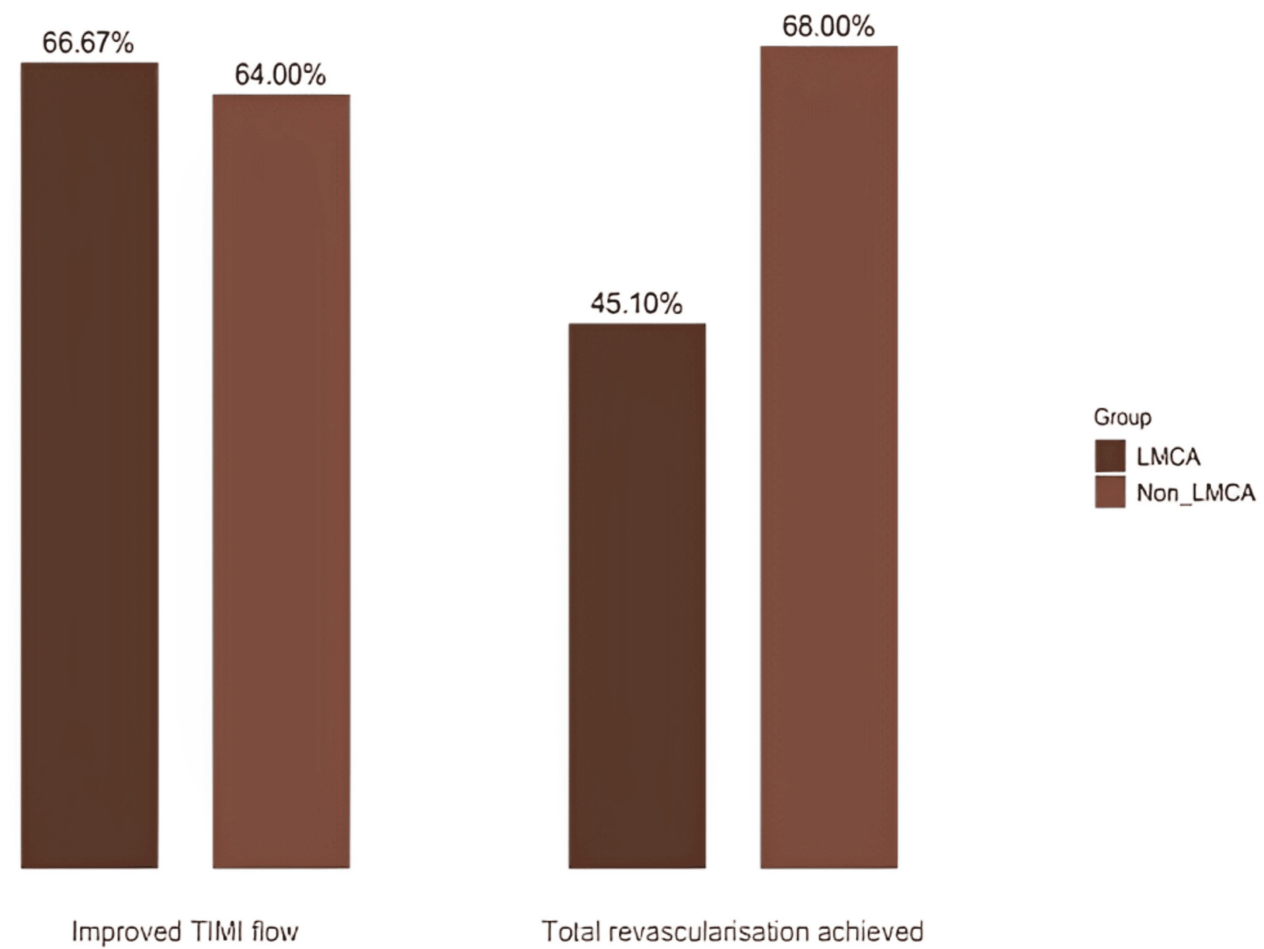
Procedural outcome of the patients (total n = 101) LMCA: Left main coronary artery, TIMI: Thrombolysis in myocardial infarction

Improved TIMI flow was achieved in 67% of the patients with LMCA disease, comparable to 64% of the patients with non-LMCA disease (p = 0.467). However, total revascularization was notably different; 45% of the patients with LMCA disease achieved complete revascularization, whereas a higher percentage of 68% was reported in the non-LMCA group (p = 0.028) (Figure 1).

The primary endpoint of the study, MACE, occurred in 5.9% of LMCA patients compared to 2% in the non-LMCA group (p = 0.013). Myocardial infarction was notably higher in the LMCA group, occurring in 4% of patients compared to none in the non-LMCA group. Both groups reported no events of stroke, and cardiac death was similar between groups at 2%. Regarding secondary endpoints, persistent angina was reported in 5.9% of the patients with LMCA versus 4% in the non-LMCA group. Persistent dyspnea was also comparable at 4% in both groups. Stent thrombosis occurred in 2% of LMCA patients, while none was reported in the non-LMCA group. Additionally, repeat revascularization was significantly higher in the LMCA group, with 4% compared to none in the non-LMCA group (Table 4).​​​​ A post-hoc power analysis based on the observed MACE rates (5.88% in the LMCA group vs. 2.00% in the non-LMCA group), sample sizes (51 and 50), and α = 0.05 showed that the study had approximately 16.6% power to detect this difference.

**Table 4:** The 12-month outcome of the patients (total n = 101) LMCA: Left main coronary artery, MACE: Major adverse cardiac events

Endpoints	LMCA, n = 51	Non-LMCA, n = 50	Test statistic (value)	p-value
Primary endpoints				
MACE	3 (5.88)	1 (2.00)	Fisher’s exact test	0.013 0.304
Myocardial infarction	2 (3.92)	0 (0.00)	Fisher’s exact test	0.487
Stroke	0 (0.00)	0 (0.00)	-	-
Cardiac death	1 (1.96)	1 (2.00)	Fisher’s exact test	0.752
Secondary endpoints				
Persistent angina	3 (5.88)	2 (4.00)	Fisher’s exact test	0.640
Persistent dyspnea	2 (3.92)	2 (4.00)	Fisher’s exact test	0.854
Stent thrombosis	1 (1.96)	0 (0.00)	Fisher’s exact test	0.091
Repeat revascularization	2 (3.92)	0 (0.00)	Fisher’s exact test	0.487

## Discussion

Our study investigated the outcomes of PCI in patients with ACS involving the LMCA compared to those with ACS related to non-LMCA lesions, revealing variations in morbidity and mortality associated with PCI following LMCA-related ACS. We found that the occurrence of MACE during the one-year follow-up period after PCI, the primary endpoint of our study, was markedly higher in patients with LMCA disease compared to those with non-LMCA lesions. In our operational definition, MACE included MI, stroke, and sudden cardiac death. We observed an increased rate of MI in the LMCA group, though the incidence of stroke and mortality was comparable in both groups. These findings align with existing evidence highlighting the poor prognosis after PCI associated with LMCA lesions [8,11,12]. This poor prognosis might be attributable to the extensive myocardial territory at risk and the complex nature of these lesions, which often involve multivessel disease and more severe coronary artery pathology [12,13].

In our study, we also observed that a higher proportion of patients in the LMCA group had multiple artery involvement and a higher SYNTAX score on admission, indicating a more severe and complex presentation of disease. Besides, the rate of successful total revascularization was significantly lower in the LMCA group (45%) compared to the non-LMCA group (68%) in our study. Incomplete revascularization may be associated with poorer outcomes and increased rates of MACE [14-17]. The higher prevalence of multivessel disease and severe calcification in the LMCA group in our study could explain the challenges faced in achieving complete revascularization, as complex lesions often necessitate more intricate procedural strategies and may be less amenable to standard approaches followed in vulnerable and high-risk ACS patients [18].

In terms of secondary outcomes, persistent angina and dyspnea were reported at comparable rates across both groups. However, the presence of persistent symptoms after PCI may indicate inadequate myocardial perfusion or complications related to the procedure, particularly in the LMCA group, where the risk of restenosis or stent thrombosis may be heightened [11]. This phenomenon was also observed in our study participants, where the incidence of stent thrombosis and repeat revascularization was notably higher in the LMCA group. These findings emphasize the importance of meticulous procedural techniques and intensive post-procedural monitoring and management, as well as long-term adherence to dual antiplatelet therapy [19-21].

Though our study provided valuable insights into the outcomes of PCI for LMCA versus non-LMCA disease, it is important to recognize several limitations. First, we conducted the study in a routine clinical practice setting using convenience sampling, which resulted in differences in baseline characteristics among the groups that could affect the outcomes. Second, the relatively small sample size from a single center may restrict the generalizability of our results. Besides, the low post-hoc power (16.6%) due to small sample size and event rates may limit the ability to detect significant differences in rare outcomes like MACE. Lastly, the follow-up period was limited to 12 months, which may hinder our ability to assess longer-term outcomes for the patients. Moreover, multivessel disease in this setting requires a staged procedure, which may not always be possible due to the socioeconomic condition of most patients in Bangladesh. Considering the procedural aspect, it is now preferred to use intravascular ultrasound (IVUS) in every case of left main PCI to assess the adequacy of lesion preparation, measure precise vessel size, and ensure optimum expansion and apposition of stents. However, in our country, most patients cannot bear the extra cost of IVUS in acute clinical settings and therefore cannot use it widely. Future research involving larger, multicenter cohorts is needed to validate our findings and investigate the effects of various procedural techniques on long-term outcomes.

## Conclusions

Our study suggests that patients with ACS involving the LMCA may experience higher rates of major adverse cardiovascular events, particularly MI and repeat revascularization, compared to non-LMCA patients. This observation likely reflects the greater complexity and severity of LMCA disease, including more extensive vessel involvement and calcification. These findings highlight the importance of careful procedural planning and close follow-up in this high-risk group. However, given the single-center design, small sample size, low event rates, and resource-related procedural limitations, these results should be interpreted cautiously. While PCI appears to be a feasible treatment option, further, larger, multicenter studies are needed to confirm these findings and identify strategies for optimizing outcomes in patients with LMCA-related ACS.
